# The Role of Daily Dialysate Calcium Exposure in Phosphaturic Hormones in Dialysis Patients

**DOI:** 10.3390/life14080964

**Published:** 2024-07-31

**Authors:** Francesca K. Martino, Valentina di Vico, Anna Basso, Laura Gobbi, Lucia Federica Stefanelli, Martina Cacciapuoti, Elisabetta Bettin, Dorella Del Prete, Giuseppe Scaparrotta, Federico Nalesso, Lorenzo A. Calò

**Affiliations:** Nephrology, Dialysis and Transplantation Unit, Department of Medicine (DIMED), University of Padova, 35128 Padua, Italy; valentina.divico@aopd.veneto.it (V.d.V.); anna.basso@aopd.veneto.it (A.B.); laura.gobbi@aopd.veneto.it (L.G.); luciafederica.stefanelli@unipd.it (L.F.S.); martina.cacciapuoti@studenti.unipd.it (M.C.); elisabetta.bettin.1@studenti.unipd.it (E.B.); dorella.delprete@unipd.it (D.D.P.); giuseppe.scaparrotta@aopd.veneto.it (G.S.); renzcalo@unipd.it (L.A.C.)

**Keywords:** peritoneal dialysis, calcium bath, PTH, FGF-23, MBD-CKD

## Abstract

Managing mineral bone disease (MBD) could reduce cardiovascular risk and improve the survival of dialysis patients. Our study focuses on the impact of calcium bath exposure in dialysis patients by comparing peritoneal dialysis patients (PD, intervention group) and hemodialysis patients (HD, control group). We assessed various factors, including calcium, phosphorus, magnesium, PTH, vitamin D 25-OH, C-terminal telopeptide (CTX), and FGF-23 levels, as well as the calcium bath six hours before the blood sample and the length of daily calcium exposure. We enrolled 40 PD and 31 HD patients with a mean age of 68.7 ± 13.6 years. Our cohort had median PTH and FGF-23 levels of 194 ng/L (Interquartile range [IQR] 130-316) and 1296 pg/mL (IQR 396-2698), respectively. We identified the length of exposure to a 1.25 mmol/L calcium bath, phosphate levels, and CTX as independent predictors of PTH (OR 0.279, *p* = 0.011; OR 0.277, *p* = 0.012; OR 0.11, *p* = 0.01, respectively). In contrast, independent predictors of FGF-23 were phosphate levels (OR 0.48, *p* < 0.001) and serum calcium levels (OR 0.25, *p* = 0.015), which were affected by the calcium bath. These findings suggest that managing dialysate calcium baths impacts phosphaturic hormones and could be a critical factor in optimizing CKD-MBD treatment in PD patients, sparking a new avenue of research and potential interventions.

## 1. Introduction

The development of mineral bone disease (MBD) is a critical issue in the chronic kidney disease (CKD) context, manifesting with an abnormal metabolism of calcium, phosphorus, parathyroid hormone (PTH), and vitamin D, impairment in bone metabolism, and soft-tissue calcifications. CKD-MBD and its management significantly impact patient survival and cardiovascular events [1].

The KDIGO CKD-MBD guidelines, endorsed by the European Renal Best Practice (ERBP), suggest a therapeutic and diagnostic strategy in all stages of CKD to avoid vascular and bone complications [2] and include the optimal phosphate, calcium, and PTH levels in patients on dialysis. Specifically, calcium and phosphate should be in the normal range and, for PTH, between two and nine times the normal range [2].

Furthermore, fibroblast growth factor-23 (FGF-23), a hormone principally produced by osteocytes/osteoblasts that inhibits renal phosphate reabsorption and vitamin D 1-25 OH production, presents elevated levels in dialysis patients. Its role in bone metabolism is known in CKD patients [3,4]. However, its clinical meaning and optimal levels in CKD patients must be fully clarified.

Current KDIGO guidelines suggest reaching optimal calcium, phosphate, and PTH levels in dialysis patients, based principally on clinical studies conducted on hemodialysis (HD) patients. The same suggestions and recommendations are extended to peritoneal dialysis (PD) patients, assuming that HD and PD patients belong to the same population. However, HD and PD patients have different profiles and characteristics, such as hydration state, urine output, prevalent age, performance status, and glucose balance. In the context of CKD-MBD, calcium bath exposure could play a pivot role, considering the length of exposure, which is intermittent in HD patients (4 h three times a week) and continuous in PD patients (varying from 7 to 24 h per day). Furthermore, another difference between the two types of RRT is in the level of calcium exposure, which in HD can vary between 1.25 mmol/L, 1.5 mmol/L, and 1.75 mmol/L, while PD has only a 1.25 mmol/L or 1.75 mmol/L dialysate calcium concentration. Finally, the calcium, phosphate, and PTH balance in CKD-MBD contexts is highly heterogeneous [5]. It depends, in fact, on a multitude of factors, such as dietary calcium and phosphate intake, treatment with different analogs of vitamin D and phosphate binders, magnesium levels, systemic inflammation state, and bone metabolism.

Given CKD-MBD’s complex and heterogeneous nature, our focus was on dialysate exposure 24 h before the blood sample. Previous studies have reported different levels of calcium, phosphate, PTH, and FGF-23 according to the dialysis type and the exposure to calcium dialysate [6,7,8]. A recent meta-analysis also suggested a significant change in serum calcium and PTH levels with varying calcium dialysate exposure levels [9]. These findings underscore the need for further research in this area, highlighting the importance and relevance of our work in unraveling the complexities of CKD-MBD.

Our study aims to uncover how differences in calcium exposure, specifically related to renal replacement therapy, impact phosphaturic hormones. Specifically, the study aims to detect how the concentration of calcium in dialysate and the duration of calcium exposure influence PTH and FGF-23 in PD and HD patients. We hope to provide crucial insights that could be pivotal in formulating an optimal and personalized therapeutic approach for managing MBD-CKD in dialysis patients. Our research underscores the significance and relevance of the differences in dialysis treatment on MBD metabolism, making it a key area for further exploration and understanding.

## 2. Materials and Methods

We performed a case-control study at the Padua University Hospital’s Nephrology, Dialysis, and Transplantation Unit.

Specifically, we enrolled all adult patients (>18 years old) who had a three-times-a-week HD regimen and PD for at least six months without changing their dialysis prescription in the last month before the study. To avoid possible bias related to the circadian rhythm of PTH production [10], we enrolled only HD patients with morning shifts and blood examinations were carried out between 7 and 8 a.m. in all cohorts.

The exclusion criteria were as follows:

- a known diagnosis of oncologic disease with bone involvement;
- chronic inflammatory bowel disease;
- primary or tertiary hyperparathyroidism or parathyroidectomy;
- the presence of a colostomy;
- concomitant therapy with bisphosphonates or denosumab.

For each patient, the following parameters were collected:

- demographic data: gender, age, and dialytic vintage;
- comorbidity such as diabetes, hypertension, and dyslipidemia;
- blood examinations: calcium, phosphorus, magnesium, PTH, vitamin D 25-OH, albumin, cholesterol, triglycerides, urea, ferritin, C-terminal telopeptide (CTX), and FGF-23. Calcium corrected by albumin levels (calciumAlb) was considered. In HD patients, the blood sample was taken at the first weekly session between 7 and 8 a.m., while in PD patients, blood samples were taken on weekdays between 7 and 8 a.m.

Third-generation assay kits from DiaSorin were run on a LIAISON^®^ analyzer (DiaSorin, Stillwater, MN 55082, USA) to quantitatively determine whole PTH (1-84) in EDTA-plasma samples.

The “LIAISON^®^ FGF-23” test, which employs a chemiluminescence immunoassay (CLIA), was used to determine intact FGF-23 polypeptides in human EDTA plasma samples.

- urine output in 24 h;
- home therapy (vitamin D and analogs, phosphorus binders, and calcium mimetics). Specifically, the dose of phosphorus binders (sevelamer carbonate, calcium carbonate, lanthanum carbonate, aluminum salts, and sucroferric oxyhydrox-idein) in the 24 h before blood collection was considered. The weekly cumulative dose for vitamin D analogs and calcium mimetics was calculated to avoid possible unevenness in the dosage assessment between HD and PD. Furthermore, the equivalent dose for calcitriol and paracalcitol was estimated, considering that 0.01 mcg/kg calcitriol corresponds to 0.04 mcg/kg paracalcitol. Instead, the following formula was used to compare the cinacalcet and etelcalcetide doses: etelcalcetide/session = 0.111 × mg cinacalcet/day + 0.96 [11].

The following features were considered to assess the impact of calcium dialysate exposure:

(1) Hours of exposure to dialysate solution in the 24 h preceding sampling;
(2) Calcium concentration of the dialysate in the 6 h preceding sampling, considering that changes in ionized calcium concentrations mainly occur in the 30–60 min after dialysis [12,13];
(3) Hours of exposure to 1.25 mmol/L calcium dialysate solution in the 24 h preceding sampling;
(4) Hours of exposure to 1.75 mmol/L calcium dialysate solution in the 24 h preceding sampling.

Specifically, HD patients were considered as a control group with no exposure to calcium baths the day before the blood sample, while PD patients may have different exposure profiles:

- no calcium bath exposure in the previous 6 h before the blood sample (CAPD with empty abdomen at night);
- calcium bath exposure (1.25 mmol/L) in the previous 6 h before the blood sample (APD without any exchange during the day, CCPD with and without icodextrin, and CAPD without overnight icodextrin);
- calcium bath exposure 1.75 mmol/L in the previous 6 h before the blood sample (CAPD with overnight icodestrin).

Sample size: Studies have evaluated the change in PTH according to calcium dialysate exposure, while there are no studies about FGF-23 levels in different settings of calcium dialysate exposure. Based on the available studies and their characteristics, we estimated the sample size according to a report about PTH. Specifically, we used the results of Verschuur LC et al., which reported PTH levels of 39.6 pmol/L (+/−28.27) and 65.85 pmol/L (+/−34.46) in patients with 1.75 and 1.25 mmol/L calcium dialysate, respectively [14].

We estimated the sample size of the study using the OpenEpi calculator (https://www.openepi.com/SampleSize/SSCohort.htm, accessed on 20 February 2023), comparing the means of PTH with a power of 80%, confidence interval (2-sided) 95%, and a ratio of the sample size equal to 1.

The sample size formulae used are as follows:

n1 = (σ12 + σ22/k) (Z1 − α/2 + Z1 − β)2/Δ2

n2 = (k × σ12 + σ22) (Z1 − α/2 + Z1 − β)2/Δ2

The notation for the formulae are as follows: n1 = sample size of Group and n2 = sample size of Group 2.

σ1 = standard deviation of Group 1, σ2 = standard deviation of Group, Δ = difference in group means, k = ratio = n2/n1, and Z1 − α/2 = two-sided Z value (e.g., Z = 1.96 for 95% confidence interval). Z1 − β = power.

We obtained a sample size of 23 patients for each group. Therefore, to avoid selection bias in our cohort, we enrolled all Monday morning HD patients and all PD patients who satisfied the inclusion and exclusion criteria.

Statistical analysis: The continuous variables were reported as median and interquartile range and mean ± standard deviation (SD) according to their distribution. The categorical variables were described as absolute numbers and percentages. We tested the normality of the variable distribution using the Shapiro–Wilk test. Student’s *t*-test, the Mann–Whitney U, and the chi-square test were used to compare continuous and categorical variables as appropriate. Univariate and multivariate back-forward stepwise linear regression was used to evaluate the impact of covariates on PTH and FGF-23 levels after the normalization of variables through Log transformation.

Statistical significance was assessed by a two-tailed test with a *p* < 0.05.

Statistical analysis was performed with the SPSS software version 28.

## 3. Results

A total of 71 patients (median age 70 years) were enrolled, 40 on PD and 31 on HD. Table 1 summarizes the baseline characteristics of the whole cohort, as well as of HD and PD patients.

A significant difference in the use of vitamin D analogs between PD and HD patients was noted. Specifically, cholecalciferol was more commonly used in PD patients (6.5% in HD vs. 67.5% in PD, *p* < 0.001), while calcitriol was more widely used in HD patients (72.2% in HD vs. 45% in PD, *p* = 0.013). In contrast, cinacalcet was used equally in the two patient groups (22.6% in HD and 25% in PD, *p* = 0.81), while only one HD patient had etelcalcetide. Regarding binders, sevelamer carbonate was the most used (67% in HD and 80% in PD, *p* = 0.45), followed by calcium carbonate (32.3% in HD and 27.5% in PD, *p* = 0.66), lanthanum carbonate (6.5% in HD versus 35% in PD, *p* < 0.001), aluminum salts (22.5% in PD versus 6.5% in HD, *p* = 0.03), and finally sucroferric oxyhydroxide, used in 15% of DP patients.

HD patients had no calcium exposure 6 *h* before the blood sample. In contrast, 70% of the PD patients were exposed to a dialysate calcium bath of 1.25 mmol/L, 12.5% to a calcium bath of 1.75 mmol/L, and 17.5% had no calcium bath exposure. PD patients had higher serum calcium levels, which directly correlate with the calcium dialysis bath and length of the 1.75 mmol/L calcium bath. However, no correlation was found between calcium levels and the equivalent weekly dose of calcitriol or calcium mimetics (Table 2).

**Figure 1:**
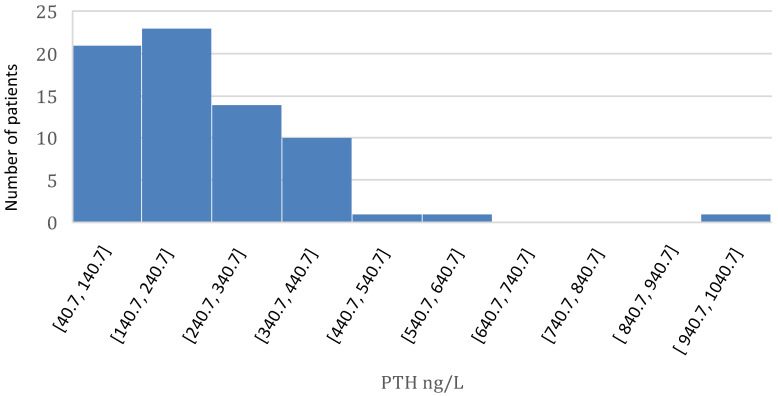
PTH distribution.

PTH distribution is reported in Figure 1. PTH levels were significantly higher in PD patients than in HD patients (269 ng/L vs. 179 ng/L, *p* < 0.006).

The PTH levels are predicted by the calcium dialysate exposure 6 h before the blood sample, the length of exposure to 1.25 mmol/L calcium dialysate solution, the phosphate and CTX levels, the calcium mimetic dose, and the patient’s age (Table 3A). Exposure length to 1.25 mmol/L calcium, phosphate, and CTX are independent predictors of PTH levels (Table 3B).

**Figure 2:**
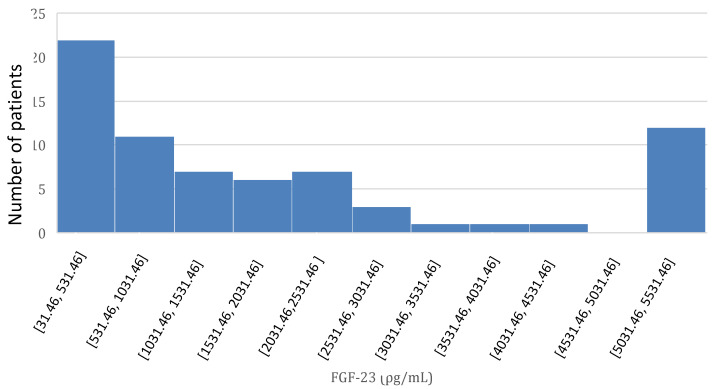
FGF-23 distribution.

FGF-23 distribution is reported in Figure 2. FGF-23 levels were significantly higher in PD patients than in HD patients (2288 pg/mL vs. 1412 pg/mL, *p* < 0.001).

FGF23 levels are predicted by the calcium exposure 6 h before the blood sample, the length of the 1.75 mmol/L calcium exposure, the level of calcium and phosphate, the cumulative dose of calcium mimetic, and the patient’s age (Table 4A).

The Model A multivariable analysis (calcium and phosphate, the cumulative dose of calcium mimetic, and the patient’s age) showed that only the phosphate and calcium levels were independent predictors of FGF23 levels (R 0.62) and Model B (calcium exposure 6 h before the blood sample, the length of 1.75 calcium exposure, phosphate level, the cumulative dose of calcium mimetic, and the patient’s age), showed that phosphate levels and calcium exposure were independent predictors (R 0.55) (Table 4B).

## 4. Discussion

The dialysate calcium bath impacts PTH and FGF-23 secretion, but while PTH secretion is independent of serum calcium levels, FGF-23 secretion is influenced by calcium levels. In our series, dialysate calcium exposure 6 h before the sample, CTX, and phosphorous levels are independent predictors of PTH. Conversely, calcium dialysate action on FGF-23 seems mediated by serum calcium levels. In a more predictive model, calcium and phosphate levels are independent predictors of FGF-23, while in a less predictive model, calcium exposure and phosphate remain independent predictors of FGF-23.

Other studies supported a relationship between PTH and calcium dialysis baths in PD [15,16,17] and HD [17,18,19], showing a significant increase in PTH with a 1.25 mmol/L calcium dialysate. Our study aimed to detect how daily calcium exposure impacts PTH and FGF-23 in PD, focusing on the presence of a calcium bath and the length of exposure. In this context, we considered HD patients, those without exposure to dialysate, and the control group belonging to the ESKD population in renal replacement treatment. Notably, despite the differences between PD and HD in age, urine output, calcium, magnesium, cholecalciferol, and calcitriol dose, only serum calcium impacts FGF-23 levels in multivariate analysis. These findings suggest a dominant role of daily calcium dialysis exposure in the secretion of PTH compared to pharmacological treatment, urine output, and patients’ age. Most likely, the prolonged exposure to calcium dialysate influences the calcium receptor set point in parathyroid cells, modulating the secretion of PTH. At the same time, the significant difference in blood calcium between HD and PD and its role as an independent predictor of FGF-23 seems to confirm the role of calcium in FGF-23 secretion reported in the animal model [20] and dialysis patients [21]. Furthermore, long daytime exposure to different calcium baths or their absence could impact circadian PTH and FGF23 secretion, which in RRT patients is poorly investigated [10,22,23] and needs to be better understood by further studies.

Our study failed to show the expected significant influence of calcium on PTH, likely due to the concomitant treatment of calcium mimetics, calcitriol, and cholecalciferol, which could impair calcium’s influence on PTH secretion. On the other hand, prolonged exposure to calcium dialysate could shape the calcium–PTH relationship [24,25]. The peculiar calcium exposure of PD could explain the lack of influence of calcium on PTH, given that the changes in calcium and not its absolute levels influence mainly PTH secretion [26]. Other studies, however, are needed to confirm this hypothesis.

Our findings corroborate the results in HD and PD regarding the effect of Cholecalciferol on FGF-23 [27,28]. On the contrary, Alshahawey et al. did not report any influence on FGF-23 after cholecalciferol supplementation in HD patients [29]. The lower prevalence of cholecalciferol treatment in HD and the different target populations could have impacted their results. However, the real influence of cholecalciferol in ESKD still needs to be fully understood.

Furthermore, our results fit with the physiologic role of PTH and FGF-23 [30,31] and previous reports about the influence of phosphate levels on phosphaturic hormones [32,33].

Differently to the findings of Corradi et al. [34], who highlighted the role of residual urine output on FGF-23 levels and found an inverse correlation between urine output and FGF-23 levels, our study did not investigate the specific meaning of urine output on the secretion of FGF-23. Corradi et al. did not demonstrate a significant impact of urine output on FGF-23 secretion in univariate and multivariate analysis, corroborating our results. However, urine output could affect FGF-23 secretion through other factors, such as phosphate levels; further studies should be carried out in this field.

Our findings suggest the potential role of dialysate calcium levels in managing CKD-MBD. Although our data need to be confirmed in a larger cohort of patients, using different calcium levels in peritoneal dialysate and various exposure lengths could help to optimize PTH and FGF-23 levels in PD patients. In the era of personalized medicine, it is crucial to recognize the unique aspects of dialysis to enhance the treatment of CKD-MBD and avoid indiscriminate pharmacological approaches in all dialysis patients. In the long term, such approaches could impact the quality of life and survival by worsening vascular calcifications and bone metabolism. The differences in PTH and FGF-23 profiles between PD and HD patients and the identification of related factors should serve as a starting point for future studies in this field. Based on our findings, we plan to evaluate the difference in the extension of vascular calcifications and bone densitometry in dialysis patients according to calcium exposure over a long period.

The present study has some limitations regarding the number of patients enrolled, which was not adequate for a definitive conclusion on FGF-23 levels. The lack of previous studies about the role of calcium dialysate and its exposure length on the levels of FGF23 did not permit us to shape the sample size for this feature. Specifically, a few studies have evaluated FGF23 levels comparing HD and PD patients [34,35], but no studies have assessed the role of calcium baths on FGF-23 levels. Our results hold attention in the context of CKD-MBD. However, other factors, such as urine toxins and hydration status, could theoretically affect the secretion of phosphaturic hormones. Nevertheless, our report is the first evidence of the influence of exposure to a calcium bath on PTH and FGF-23. From this perspective, our research group is currently conducting preliminary evaluations to determine the sample size based on our findings. This will enable us to demonstrate the impact of calcium dialysate on FGF-23 levels and to validate the results of our current study on FGF-23. We will also explore other potential factors that may affect the secretion of PTH and FGF-23. Furthermore, we did not detect the calcium and phosphate intake by dietary diary and their excretion in urine and peritoneal effluent. However, serum calcium and phosphate levels play a leading role in PTH and FGF-23 homeostasis [3]. A possible difference in calcium and phosphate intake and excretion should primarily influence serum calcium and phosphate levels and, only indirectly, PTH and FGF-23 levels. Thirdly, we did not check ionized calcium levels, which could influence calcium balance and PTH and FGF-23 secretion. However, we corrected calcium by albumin levels to reduce a possible gap. Finally, the PD and HD groups exhibited disparities in diabetes prevalence, dialysis vintage, urine output, and vitamin D supplementation, which could potentially impact our findings. However, our univariate analysis did not indicate a significant role for these factors in our PTH and FGF23 secretion model.

## 5. Conclusions

For the first time, our study demonstrates how the presence of and length of exposure to calcium dialysate impact phosphaturic hormones. The length of exposure to calcium dialysate independently predicts PTH level, while FGF-23 seems to be influenced by calcium exposure through serum calcium. These findings suggest that managing dialysate calcium baths in PD patients is a therapeutic option for treating CKD-MBD, like vitamin D analogs and calcium mimetics. However, it is crucial to emphasize that further experimental and clinical studies are definitively needed to confirm this working hypothesis.

## 6. Patents

Our study focused on adult patients (>18 years old) treated with renal replacement therapy who had PD treatment or a three-times-a-week HD regimen. All patients had at least six months of dialysis without changing dialysis prescriptions in the last month before the study. It is important to reiterate that the patients with the presence of clinical conditions that can impair calcium or phosphate metabolism (such as bone oncologic lesions, chronic inflammatory bowel disease or presence of colostomy, primary or tertiary hyperparathyroidism or parathyroidectomy, and concomitant therapy with bisphosphonates or denosumab) were strictly excluded.

## Figures and Tables

**Table 1:** Baseline characteristics of the whole population, PD patients, and HD patients.

Parameter	Total (=71)	Peritoneal Dialysis (=40)	Hemodialysis (=31)	*p*
Male (n%)	41 (57.7%)	13 (31.7%)	17 (54.8%)	0.059
Diabetes (n%)	23 (32.4%)	9 (22.5%)	14 (45.1%)	0.043
Hypertension (n%)	64 (90.1%)	35 (87.5%)	29 (93.5%)	0.39
Age (years)	68.7 (±13.6)	65.9 (±13.3)	72.2 (±13.2)	0.053
Dialytic Vintage (years)	3 (2–4)	2 (2–3)	4 (2–6)	0.009
Urine out-put (cc/d)	800 (300–1300)	1000 (500–1575)	600 (0–830)	0.006
Calcium_Alb_ (mmol/L)	2.33 (±0.19)	2.39 (±0.17)	2.26 (±0.2)	0.009
Phosphate (mmol/L)	1.5 (1.32–1.81)	1.59 (1.3–1.8)	1.45 (1.3–1.7)	0.63
Magnesium (mmol/L)	0.91 (±0.19)	0.99 (±0.18)	0.8 (±0.18)	<0.001
PTH (ng/L)	194 (130–316)	238 (162–355)	159 (100–229)	0.006
FGF-23 (pg/mL)	1296 (396–2698)	1630 (521–3976)	780 (389–1806)	0.048
CTX (pg/mL)	2336 (1402–4525)	2341 (1614–4558)	2336 (1364–4525)	0.56
Haemoglobin (g/L)	107 (98–118)	108 (99.7–120)	107 (94–117)	0.65
Albumin (g/L)	33.5 (±4.6)	33.6 (±4.3)	33.4 (±4.9)	0.83
Cholesterol (mg/dL)	147 (117–174)	145 (114–172)	147 (121–176)	0.82
Triglycerides (mg/dL)	121 (86–166)	121 (81–183)	121 (89–155)	0.76
Urea (mmol/L)	20.3 (16.4–22.9)	18.6 (16.4–22.5)	21.9 (16.5–23.7)	0.14
Kt/V	NA	2.08 (1.78–2.4)	1.2 (1.06–1.35)	NA
Ca-Dialysate (mmol/L)	1.25 (0–1.25)	1.25 (1.25–1.25)	0 (0–0)	<0.001
_1.25_ CaExp (hours)	7 (0–9)	9 (8.1–12)	0 (0–0)	<0.001
_1.75_ CaExp (hours)	0 (0–10)	4 (0–14)	0 (0–0)	<0.001
Cholecalciferol dose (UI/week)	0 (0–5000)	5000 (0–6250)	0 (0–0)	<0.001
Calcitriol equivalent dose (mcg/week)	1.75 (1.25–3.75)	1.75 (0–1.75)	3.25 (3.5–3.75)	0.007
Cinacalcet equivalent dose (mg/week)	0 (0–0)	0 (0–78.7)	0 (0–0)	0.62
Calcium based binder dose (g/d)	0 (0–1)	0 (0–1)	0 (0–1)	0.75

Footnote: calcium corrected by albumin (CalciumAlb), calcium exposure in the 6 h before the blood sample (Ca-Dialysate), hours of exposure to 1.25 mmol/L calcium dialysate in 24 h (1.25 CaExp), hours of exposure to 1.75 mmol/L calcium dialysate in 24 h (1.75CaExp), parathormone (PTH), and C-terminal telopeptide (CTX).

**Table 2:** Relationship between serum calcium levels and calcium dialysis features.

	Rho	*p*
Ca-Dialysate (mmol/L)	0.41	0.008
_1.25_ CaExp (h)	0.35	0.003
_1.75_ CaExp (h)	0.27	0.025
Cholecalciferol dose (UI/week)	0.36	0.002
Calcitriol equivalent dose (mcg/week)	0.54	0.66
Cinacalcet equivalent dose (mg/week)	0.03	0.81
Ca-Based P binder dose (g/d)	−0.031	0.80

Footnote: Spearman correlation. Calcium exposure in the 6 h before the blood sample (Ca-Dialysate), hours of exposure to 1.25 mmol/L calcium dialysate in 24 h (1.25 CaExp), and hours of exposure to 1.75 mmol/L calcium dialysate in 24 h (1.75CaExp).

**Table 3:** PTH level predictors in univariate (**A**) and multivariate (**B**) linear regression analysis.

**A. Univariate Regression Analysis**			
	**Beta-Coefficient**	***p***	**95% IC**
Age	−0.25	0.038	−0.02 −0.021
Urine output	0.116	0.33	−0.02 0.00
Ca-Dialysate	0.29	0.014	0.05 0.45
_1.25_ CaExp	0.25	0.039	0.001 0.05
_1.75_ CaExp	−0.28	0.82	−0.3 0.2
Calcium_Alb_	−0.12	0.31	−1.12 0.36
Phosphate	0.28	0.016	0.8 0.76
Magnesium	0.18	0.13	−0.16 1.31
FGF-23 (pg/mL)	0.12	0.3	−0.00 0.00
CTX (pg/mL)	0.3	0.011	0.00 0.00
Cholecalciferol dose	0.03	0.82	0.00 0.00
Calcitriol equivalent dose	−0.11	0.35	−0.15 0.05
Cinacalcet equivalent dose	0.25	0.04	0.00 0.003
Ca-Based P binder Dose	0.12	0.31	−0.77 0.24
**B. Multivariate Regression Analysis**			
_1.25_ CaExp	0.279	0.011	0.05 0.10
P (mmol/L)	0.277	0.012	0.09 0.7
CTX (pg/mL)	0.29	0.009	0.00 0.00

Footnote: calcium corrected by albumin (CalciumAlb), calcium exposure in the 6 h before the blood sample (Ca-Dialysate), hours of exposure to 1.25 mmol/L calcium dialysate in 24 h (1.25 CaExp), hours of exposure to 1.75 mmol/L calcium dialysate in 24 h (1.75CaExp), parathormone (PTH), and C-terminal telopeptide (CTX).

**Table 4:** FGF23 level predictors in univariate (**A**) and multivariate (**B**) linear regression analysis.

**A. Univariate Regression Analysis**			
	**Beta-Coefficient**	***p***	**95% IC**
Age	−0.37	0.002	−0.06 −0.01
Urine output	0.08	0.48	−0.00 0.00
Ca-Dialysate	0.26	0.027	0.06 0.95
Exp_tot_	0.30	0.68	6.8 48
_1.25_ CaExp	0.19	0.10	−0.009 0.09
_1.75_ CaExp	0.21	0.08	−0.006 0.09
Calcium_Alb_	0.26	0.026	0.22 3.4
Phosphorous	0.49	<0.001	0.92 2.28
Magnesium	0.29	0.013	0.43 3.6
PTH (ng/L)	−0.006	0.96	−0.002 0.002
CTX (pg/mL)	−0.09	0.43	−0.00 0.00
Cholecalciferol (UI/week)	0.16	0.18	0.00 0.00
Calcitriol equivalent dose (mcg/week)	−0.063	0.6	−0.28 0.16
Cinacalcet equivalent dose (mg/week)	0.27	0.021	0.001 0.007
Ca-based P binder dose (g/d)	−0.10	0.39	−0.49 0.2
**B. Multivariate Regression Analysis**			
	Model A: R = 0.62		
Phosphorous	0.57	<0.001	1.24 2.5
CalciumAlb	0.38	<0.001	1.32 3.98
	Model B: R = 0.55		
Phosphorous	0.48	<0.001	0.93 2.2
Ca-Dialysate	0.25	0.015	0.09 0.87

Footnote: calcium corrected by albumin (CalciumAlb), calcium exposure in the 6 h before the blood sample (Ca-Dialysate), hours of exposure to 1.25 mmol/L calcium dialysate in 24 h (1.25 CaExp), hours of exposure to 1.75 mmol/L calcium dialysate in 24 h (1.75CaExp), parathormone (PTH), and C-terminal telopeptide (CTX).

## Data Availability

The data presented in this study are available on request from the corresponding author. The data are not publicly available due to privacy.
